# Supplementary material for: Direct tissue-sensing reprograms TLR4+ Tfh-like cells inflammatory profile in the joints of rheumatoid arthritis patients
Source: Commun Biol. 2021 Sep 27;4:1135. doi: 10.1038/s42003-021-02659-0 (PMC8476501; doi:10.1038/s42003-021-02659-0)
Supplement: Supplementary file 2 — Supplementary Information [file 42003_2021_2659_MOESM2_ESM.pdf]

a

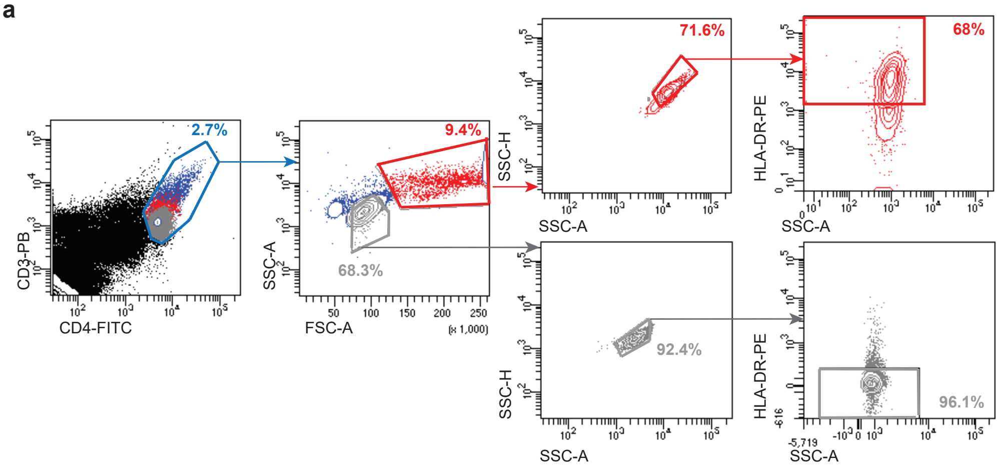

Supplementary Figure 1. FACS-purification strategy and sorted cell population purity. a Flow cytometric sorting strategy for the purification of CD3^{high}CD4^{high}HLA-DR^{+} and CD3^{high}CD4^{high}HLA-DR^{-} T cells.

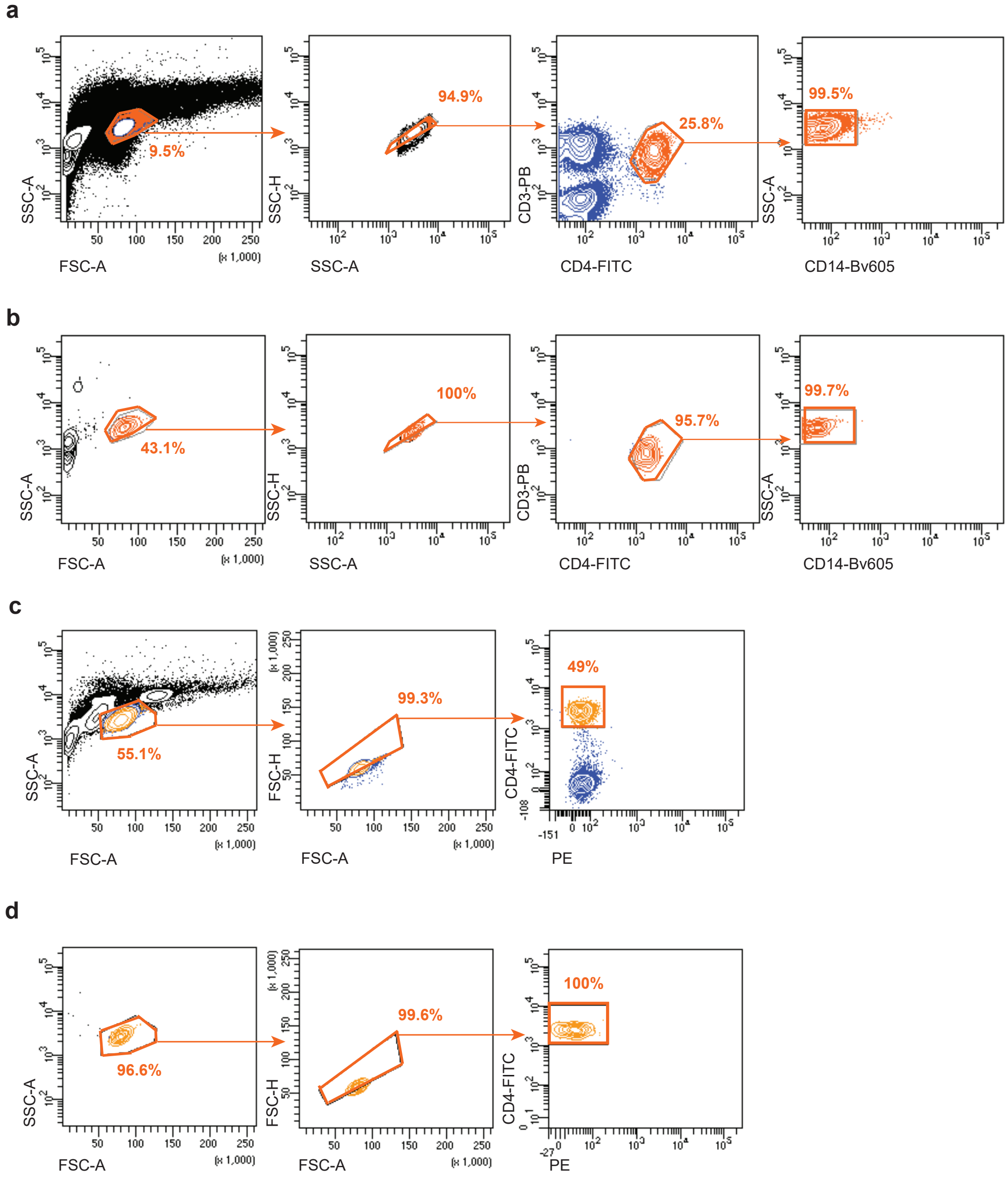

Supplementary Figure 2. FACS-purification strategy and sorted cell population purity of RA patient and healthy donor. a Flow cytometric sorting strategy for the purification of CD14^{-} CD3^{+}CD4^{+}SSC^{low}FSC^{low}T cells from RA patients. **b** Purity of sorted CD14^{-}CD3^{+}CD4^{+}SSC^{low}FSC^{low}T cells from RA patients. **c** Flow cytometric sorting strategy for the purification of CD3^{high}CD4^{high}FSC^{low}SSC^{low} T cells from healthy donor. **d** Purity of sorted CD3^{high}CD4^{high}FSC^{low}SSC^{low} T cells from healthy donor.

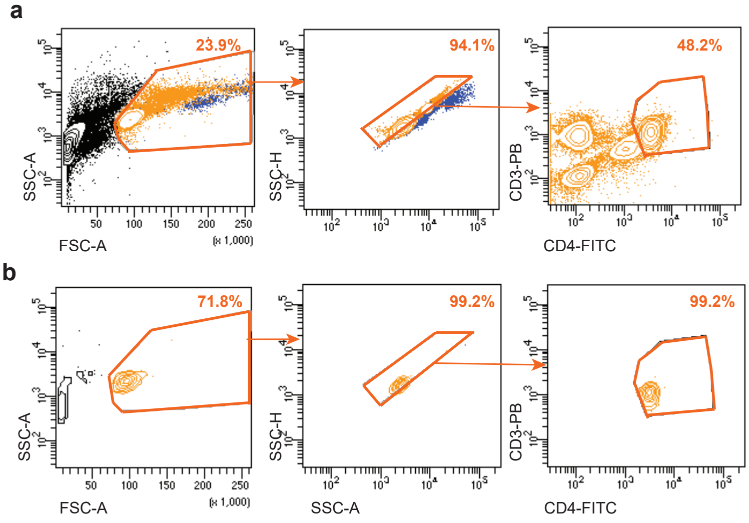

Supplementary Figure 3. FACS-purification strategy for CD4⁺T cells from RA patients. a Flow cytometric sorting strategy for the purification of $CD3^{high}CD4^{high}$ T cells. **b** Purity of sorted $CD3^{high}CD4^{high}$ T cells.

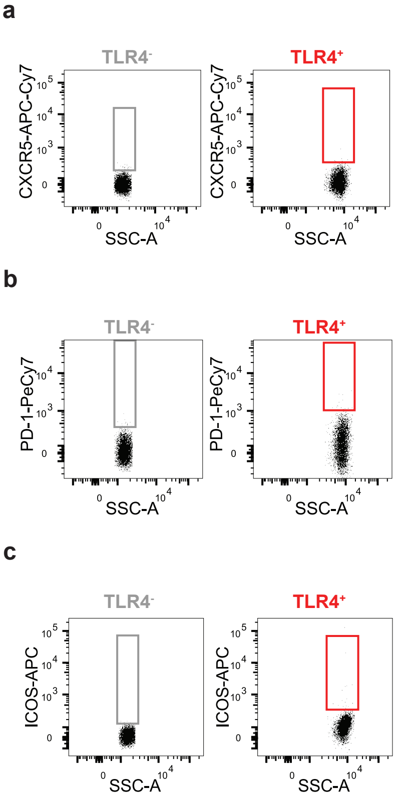

Supplementary Figure 4. Fluorescence minus one (FMO) conditions for gating Tfh markers in TLR4$^-$ and TLR4$^+$ T cell populations. a Representative FMO dot plot for CXCR5 in TLR4$^-$ (grey) versus TLR4$^+$ (red) T cells, with gating illustrating the individual positivity cut-off for both cell populations. **b** Representative FMO dot plot for PD-1 in TLR4$^-$ (grey) versus TLR4$^+$ (red) T cells, with gating illustrating the individual positivity cut-off for both cell populations. **c** Representative FMO dot plot for ICOS in TLR4$^-$ (grey) versus TLR4$^+$ (red) T cells, with gating illustrating the individual positivity cut-off for both cell populations.

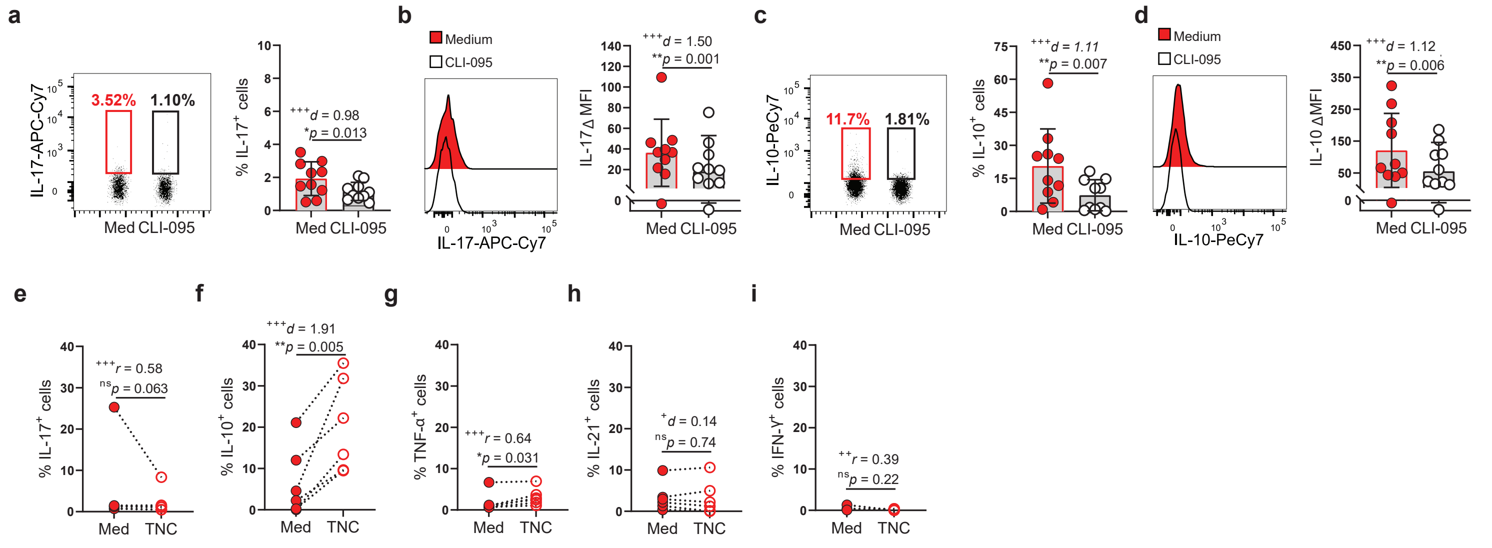

Supplementary Figure 5. Ongoing cytokine production by circulating TLR4⁺ T cells is due to TLR4 engagement. a-d FACS-purified CD3^{high}CD4^{high} T cells from peripheral blood were cultured for 18 hours in the presence of medium (Med) or TLR4 signaling inhibitor (CLI-095). Frequency and ΔMFI of IL-17 (**a, b**) and IL-10 (**c, d**) production by TLR4⁺ T cells (*n* = 10 RA patients). **e-i** FACS-purified CD3^{high}CD4^{high} T cells from peripheral blood were cultured for 18 hours in the presence of medium (Med) or with TLR4 endogenous ligand tenascin-C (TNC). Frequency of **e** IL-17, **f** IL-10, **g** TNF-α, **h** IL-21 and **i** IFN-γ production by TLR4⁺ T cells (*n* = 6 RA patients). ΔMFI was calculated by subtracting the fluorescence intensity minus one (FMO) from median fluorescence intensity (MFI) for each given marker. Data is presented as mean ± SD, for parametric statistical tests, or median ± IQR, for non-parametric statistical tests. Sample normality distribution was tested by using D'Agostino & Pearson normality test (n>6) or Shapiro-Wilk normality test (n≤6). *P*-values *****p* ≤ 0.0001, ****p* ≤ 0.001, ***p* ≤ 0.01, **p* ≤ 0.05 were determined by (a, b, c, d, f, h) Paired t-test and (e, g, i) Wilcoxon matched-pairs rank test. (m, o, p). Effect size measures $^{+++}$high, $^{++}$medium, $^{+}$small were determined by (a, b, c, d, f, h) *d* - Cohen's *d* and (e, g, i) *r* - correlation coefficient *r*.

Supplementary Table 1. Demographic and experiments with healthy donors

Subject ID	Age	Gender	Blood Phenotyping	Confocal Microscopy
CEDOC_HD_001	58	F	+	
CEDOC_HD_002	55	F	+	
CEDOC_HD_003	24	M	+	
CEDOC_HD_004	41	F	+	
CEDOC_HD_005	28	F	+	
CEDOC_HD_006	66	F	+	
CEDOC_HD_007	29	F	+	+
CEDOC_HD_008	54	F	+	
CEDOC_HD_009	55	F	+	
CEDOC_HD_010	25	F	+	
CEDOC_HD_011	27	F	+	
CEDOC_HD_012	28	F	+	
CEDOC_HD_013	23	F	+	
CEDOC_HD_014	29	F	+	
CEDOC_HD_015	47	F	+	
CEDOC_HD_016	24	F	+	
CEDOC_HD_017	28	F	+	
CEDOC_HD_018	22	F	+	
CEDOC_HD_019	23	F	+	
CEDOC_HD_020	24	F	+	
CEDOC_HD_021	23	F	+	+
CEDOC_HD_022	42	F	+	
CEDOC_HD_023	30	F	+	
CEDOC_HD_024	24	F	+	
CEDOC_HD_025	66	F	+	
CEDOC_HD_026	39	F	+	
CEDOC_HD_027	31	F	+	+
CEDOC_HD_028	28	F	+	
CEDOC_HD_029	25	F	+	+
CEDOC_HD_030	30	F	+	

F – Female

M – Male

+ – performed assay
