# Supplementary material for: Direct tissue-sensing reprograms TLR4+ Tfh-like cells inflammatory profile in the joints of rheumatoid arthritis patients
Source: Commun Biol. 2021 Sep 27;4:1135. doi: 10.1038/s42003-021-02659-0 (PMC8476501; doi:10.1038/s42003-021-02659-0)
Supplement: Supplementary file 3 — Description of Supplementary Files [file 42003_2021_2659_MOESM3_ESM.pdf]

Description of Supplementary Files

File name: Supplementary Data 1
Description: Contain demographic, clinical data and experiments with RA Patients

File name: Supplementary Data 2
Description: Contains list of reagents and software

File name: Supplementary Data 3
Description: Contains statistical summary per figure

File name: Supplementary Data 4
Description: Contains all the raw data used in Figure 1

File name: Supplementary Data 5
Description: Contains all the raw data used in Figure 2

File name: Supplementary Data 6
Description: Contains all the raw data used in Figure 3

File name: Supplementary Data 7
Description: Contains all the raw data used in Figure 4

File name: Supplementary Data 8
Description: Contains all the raw data used in Figure 5

File name: Supplementary Data 9
Description: Contains all the raw data used in Figure 6

File name: Supplementary Data 10
Description: Contains all the raw data used in Figure 7

File name: Supplementary Data 11
Description: Contains all the raw data used in Figure 8

File name: Supplementary Data 12
Description: Contains all the raw data used in Figure 9

File name: Supplementary Data 13
Description: Contains all the raw data used in Figure 10

File name: Supplementary Data 14
Description: Contains all the raw data used in Supplementary Figure 5
