# Supplementary material for: Direct tissue-sensing reprograms TLR4+ Tfh-like cells inflammatory profile in the joints of rheumatoid arthritis patients
Source: Commun Biol. 2021 Sep 27;4:1135. doi: 10.1038/s42003-021-02659-0 (PMC8476501; doi:10.1038/s42003-021-02659-0)
Supplement: Supplementary file 4 — Supplementary Data 1 [file 42003_2021_2659_MOESM4_ESM.docx]

Supplementary Data 1. Demographic, clinical data and experiments with RA Patients

| Subject ID | Age | Gender | Blood Phenotyping | Synovial Fluid Phenotyping | Receptors | Cytokines | Anti-CCP ELISA | Confocal Microscopy |
| --- | --- | --- | --- | --- | --- | --- | --- | --- |
| HEM_RA_016 | 49 | F | + |  |  |  | + |  |
| HEM_RA_032 | 38 | F | + |  |  |  | + |  |
| HEM_RA_011 | 71 | F | + |  |  |  | + |  |
| HEM_RA_036 | 48 | F | + |  |  |  | + |  |
| HEM_RA_037 | 59 | F | + |  |  |  | + |  |
| HEM_RA_038 | 57 | F | + |  |  |  | + |  |
| HEM_RA_023 | 40 | F | + |  |  |  | + |  |
| HEM_RA_030 | 54 | M | + |  |  |  | + |  |
| HEM_RA_007 | 76 | F | + |  |  |  | + |  |
| HEM_RA_001 | 60 | F | + |  |  |  | + |  |
| HEM_RA_039 | 54 | F | + |  |  |  | + |  |
| HEM_RA_063 | 66 | F | + |  |  |  | + |  |
| HEM_RA_064 | 55 | M | + |  |  |  | + |  |
| HEM_RA_065 | 59 | F | + |  |  |  | + |  |
| HEM_RA_066 | 68 | M | + |  |  |  | + |  |
| HEM_RA_067 | 70 | F | + |  |  |  | + |  |
| HEM_RA_051 | 61 | F | + | + |  |  | + |  |
| HEM_RA_068 | 65 | M | + | + |  |  | + |  |
| HEM_RA_069 | 61 | M | + | + |  |  | + |  |
| HEM_RA_070 | 72 | F | + | + |  |  | + |  |
| HEM_RA_071 | 58 | F | + |  |  |  | + |  |
| HEM_RA_072 | 61 | F | + |  |  |  | + |  |
| HEM_RA_073 | 73 | F | + |  |  |  | + |  |
| HEM_RA_074 | 82 | F | + |  |  |  | + |  |
| HEM_RA_075 | 41 | F | + |  |  |  | + |  |
| HEM_RA_076 | 62 | F | + |  |  |  | + |  |
| HEM_RA_078 | 70 | F | + |  |  |  | + |  |
| HEM_RA_079 | 73 | F | + |  |  |  | + |  |
| HEM_RA_080 | 79 | F | + |  |  |  | + |  |
| HEM_RA_081 | 71 | F | + |  |  |  | + |  |
| HEM_RA_082 | 43 | F | + |  |  |  | + |  |
| HEM_RA_083 | 50 | F | + |  |  |  | + |  |
| HEM_RA_084 | 78 | F | + |  |  |  | + |  |
| HEM_RA_085 | 61 | F | + |  |  |  | + |  |
| HEM_RA_086 | 51 | F | + |  |  |  | + |  |
| HEM_RA_087 | 85 | F | + |  |  |  | + |  |
| HEM_RA_088 | 65 | F | + |  |  |  | + |  |
| HEM_RA_089 | 60 | F | + |  |  |  | + |  |
| HEM_RA_090 | 52 | F | + |  |  |  | + |  |
| HEM_RA_091 | 80 | F | + |  |  |  | + |  |
| HEM_RA_092 | 78 | F | + |  |  |  | + |  |
| HEM_RA_093 | 60 | F | + | + |  |  | + |  |
| HEM_RA_094 | 72 | F | + |  |  |  | + |  |
| HEM_RA_095 | 49 | F | + |  |  |  | + |  |
| HEM_RA_096 | 55 | F | + |  |  |  | + |  |
| HEM_RA_097 | 68 | F | + |  |  | + | + |  |
| HEM_RA_098 | 80 | F | + |  |  | + | + |  |
| HEM_RA_099 | 46 | M | + |  |  | + | + |  |
| HEM_RA_100 | 85 | F | + |  |  |  | + |  |
| HEM_RA_101 | 63 | F | + |  |  |  | + |  |
| HEM_RA_102 | 50 | F | + |  |  | + | + |  |
| HEM_RA_103 | 42 | F | + |  |  | + | + |  |
| HEM_RA_104 | 62 | F | + |  |  | + | + |  |
| HEM_RA_105 | 57 | F | + |  |  | + | + |  |
| HEM_RA_106 | 82 | F | + |  |  |  | + |  |
| HEM_RA_107 | 72 | F | + |  |  |  | + |  |
| HEM_RA_108 | 60 | F | + |  |  |  | + |  |
| HEM_RA_109 | 52 | F | + |  |  |  | + |  |
| HEM_RA_110 | 68 | F | + |  |  | + | + |  |
| HEM_RA_111 | 61 | F | + |  |  | + | + |  |
| HEM_RA_112 | 85 | F | + |  |  | + | + |  |
| HEM_RA_113 | 71 | F | + |  |  | + | + |  |
| HEM_RA_114 | 56 | F | + |  |  |  | + |  |
| HEM_RA_115 | 60 | F | + |  |  |  | + |  |
| HEM_RA_116 | 42 | M | + |  |  |  | + |  |
| HEM_RA_117 | 56 | M | + |  |  |  | + |  |
| HEM_RA_118 | 53 | F | + |  |  | + | + |  |
| HEM_RA_119 | 45 | F | + |  |  | + | + |  |
| HEM_RA_120 | 62 | F | + |  |  | + | + |  |
| HEM_RA_123 | 31 | F | + |  |  |  | + |  |
| HEM_RA_124 | 78 | F | + |  |  | + | + |  |
| HEM_RA_125 | 55 | F | + |  |  | + | + |  |
| HEM_RA_126 | 67 | F | + |  |  | + | + |  |
| HEM_RA_127 | 63 | F | + |  |  |  | + | + |
| HEM_RA_128 | 60 | F | + |  | + |  | + |  |
| HEM_RA_129 | 76 | M | + |  | + | + | + |  |
| HEM_RA_130 | 42 | M | + |  |  | + | + |  |
| HEM_RA_131 | 78 | M | + |  | + |  | + |  |
| HEM_RA_132 | 64 | M | + |  | + |  | + |  |
| HEM_RA_133 | 47 | F | + |  | + |  | + |  |
| HEM_RA_134 | 57 | F | + | + |  |  | + |  |
| HEM_RA_135 | 77 | F | + |  |  | + | + |  |
| HEM_RA_136 | 76 | F | + |  |  | + | + |  |
| HEM_RA_137 | 64 | F | + |  | + |  | + |  |
| HEM_RA_138 | 66 | F | + |  | + |  | + |  |
| HEM_RA_139 | 40 | M | + |  |  | + | + |  |
| HEM_RA_140 | 56 | M | + |  | + |  | + |  |
| HEM_RA_141 | 54 | F | + |  | + | + | + |  |
| HEM_RA_142 | 48 | F | + |  | + |  | + |  |
| HEM_RA_143 | 48 | F | + |  |  | + | + |  |
| HEM_RA_144 | 59 | F | + |  |  | + | + |  |
| HEM_RA_145 | 72 | F | + |  | + | + | + |  |
| HEM_RA_146 | 69 | F | + |  | + |  | + |  |
| HEM_RA_147 | 65 | F | + |  | + |  | + |  |
| HEM_RA_148 | 74 | F | + |  |  | + | + |  |
| HEM_RA_149 | 37 | F | + |  |  | + | + |  |
| HEM_RA_150 | 36 | F | + | + |  |  | + |  |
| HEM_RA_151 | 40 | F | + |  |  | + |  |  |
| HEM_RA_152 | 72 | F | + |  |  | + |  |  |
| HEM_RA_153 | 64 | F | + |  |  | + |  |  |
| HEM_RA_154 | 68 | F | + |  |  | + |  |  |
| HEM_RA_155 | 52 | M | + | + |  | + |  |  |
| HEM_RA_156 | 72 | F | + | + |  | + |  |  |
| HEM_RA_157 | 61 | F |  |  |  | + |  |  |
| HEM_RA_158 | 61 | F | + | + |  |  |  |  |
| HEM_RA_159 | 57 | F |  |  |  | + |  |  |
| HEM_RA_160 | 86 | F | + | + |  | + |  |  |
| HEM_RA_161 | 57 | F | + |  |  |  |  |  |
| HEM_RA_162 | 44 | F | + |  |  |  |  |  |
| HEM_RA_163 | 63 | F | + |  |  |  |  |  |
| HEM_RA_164 | 81 | M | + | + |  |  |  |  |
| HEM_RA_165 | 77 | F | + |  |  |  |  |  |
| HEM_RA_166 | 49 | F |  |  |  | + |  |  |
| HEM_RA_167 | 69 | M | + |  |  |  |  |  |
| HEM_RA_168 | 75 | F | + |  |  |  |  |  |
| HEM_RA_169 | 78 | M |  |  |  | + |  |  |
| HEM_RA_173 | 55 | F |  |  |  | + |  |  |
| HEM_RA_174 | 55 | F |  |  |  | + |  |  |
| HEM_RA_175 | 60 | M |  |  |  | + |  |  |
| HEM_RA_176 | 65 | M |  |  |  | + |  |  |

|  |
| --- |

F – Female

M – Male

+ – performed assay
