# Supplementary material for: Direct tissue-sensing reprograms TLR4+ Tfh-like cells inflammatory profile in the joints of rheumatoid arthritis patients
Source: Commun Biol. 2021 Sep 27;4:1135. doi: 10.1038/s42003-021-02659-0 (PMC8476501; doi:10.1038/s42003-021-02659-0)
Supplement: Supplementary file 5 — Supplementary Data 2 [file 42003_2021_2659_MOESM5_ESM.docx]

Supplementary Data 2. List of reagents and software

| Reagent or Resource | Source | Identifier |
| --- | --- | --- |
| Antibodies | | |
| Anti-mouse IgG1 | BioLegend | Cat#406602 |
| Anti-hamster IgG | Thermo Fisher Scientific | Cat#31115 |
| Anti-CD3 (UCHT1) | BioLegend | Cat#300402 |
| Anti-HLA-DR (L243) | BioLegend | Cat#307602 |
| Anti-ICOS (C398-4A) | BioLegend | Cat#313512 |
| Anti-TLR4 (HTA125) | BioLegend | Cat#312804 |
| Anti-TLR4 (76B357.1) | Abcam | Cat#ab22048 |
| Anti-IL1R (C-20) | Santa Cruz | Cat#sc-687 |
| Anti-CD28 (CD28.2) | BioLegend | Cat#302914 |
|  |  |  |
| Anti-CD4 (RPA-T4) | BioLegend | Cat#300506 (FITC) |
| Anti-HLA-DR (L243) | BioLegend | Cat#307606 (PE) |
| Anti-TLR4 (HTA125) | BioLegend | Cat#312805 (PE) |
| Streptavidin | Biolegend | Cat#405205 (PeCy5) |
| Anti-TNF-α (MAb11) | Biolegend | Cat#502926 (PerCP/Cy5.5) |
| Anti-PD1 (EH12.2H7) | BioLegend | Cat#329917 (PeCy7) |
| Anti-CCR2 (K036C2) | BioLegend | Cat#357211 (PeCy7) |
| Anti-CD25 (M-A251) | BioLegend | Cat#356107 (PeCy7) |
| Anti-Ki67 (B56) | BD Pharmigen | Cat#561283 (PeCy7) |
| Anti-TNFa (MAb11) | BioLegend | Cat#502929 (PeCy7) |
| Anti-IL-10 (JES3-9D7) | BioLegend | Cat#501419 (PeCy7) |
| Anti-CD4 (RPA-T4) | BioLegend | Cat#300514 (APC) |
| Anti-IL6R (UV4) | BioLegend | Cat#352805 (APC) |
| Anti-ICOS (C398.4A) | BioLegend | Cat#313510 (APC) |
| Anti-IL17R (BG/hIL17AR) | BioLegend | Cat#340903 (A647) |
| Anti-IL-21 (3A3-N2) | BioLegend | Cat#513006 (A647) |
| Anti-rabbit | Invitrogen | Cat#A-21244 (A647) |
| Anti-mouse IgG1 | Thermo Fisher | Cat#A21240 (A647) |
| Anti-CD3 (HIT3a) | BioLegend | Cat#300318 (APC-Cy7) |
| Anti-CCR6 (G034E3) | BioLegend | Cat#353432 (APC-Cy7) |
| Anti-CD38 (HIT2) | BioLegend | Cat#303533 (APC-Cy7) |
| Anti-CXCR5 (J252D4) | BioLegend | Cat#356925 (APC-Cy7) |
| Anti-IL17 (BL168) | Biolegend | Cat#512320 (APC-Cy7) |
| Anti-CD14 (63D3) | BioLegend | Cat#367107 (APC-Cy7) |
|  |  |  |
| Anti-CD3 (SK7) | BioLegend | Cat#344828 (Bv510) |
| Anti-CD3 (SK7) | BioLegend | Cat#3448284 (PB) |
| Anti-IFN-γ (4S.B3) | BioLegend | Cat#502522 (PB) |
|  |  |  |
| Anti-mouse IgG2b | Thermo Fisher | Cat#A21141 (A488) |
| Anti-CD4 (SK3) | BioLegend | Cat#344666 (PE-Fire 700) |
|  |  |  |
| Anti-CD3 (UCHT1) | BioLegend | Cat#300424 (A700) |
|  |  |  |
| Anti-CD14 (63D3) | BioLegend | Cat#367125 (Bv605) |
|  |  |  |
| Anti-IL-10 (JES3-19F1) | BioLegend | Cat#506811 (PE-Dazzle 594) |
|  |  |  |

Dyes

| Calcein Violet-AM | BioLegend | Cat#425203 |
| --- | --- | --- |
| Fixable Viability Dye eFluor™ 506 | eBioscience | Cat#65-0866-14 |
| Fixable Viability Dye eFluor™ 780 | eBioscience | Cat#65-0865-14 |
| Cell Trace™ Violet | Thermo Fisher | Cat#C34557 |
|  |  |  |

Chemicals

| Phosphate-buffered saline (PBS) 10x, Sterile Ultra-Pure Grade | VWR | Cat#97063-660 |
| --- | --- | --- |
| Phosphate-buffered saline (PBS) 10x, pH 7.4 | VWR | Cat#J62036.K7 |
| Biocoll | Merck Millipore | Cat#L-6715 |
| 10x RBC lysis buffer | eBioscience | Cat#00-4300-54 |
| Hyaluronidase | Sigma-Aldrich | Cat#37326-33-3 |
| Paraformaldehyde | Sigma-Aldrich | Cat#P6148 |
| eBioscience™ Foxp3 / Transcription Factor Staining Buffer Set | eBioscience | Cat#00-5523-00 |
| Saponin | Carl Roth | Cat#4185.1 |
| RPMI 1640 medium | Gibco | Cat#21875034 |
| Fetal Bovine Serum (FBS) superior | Sigma-Aldrich | Cat#S0615 |
| Antibiotic Antimycotic (100x) | Gibco | Cat#15240062 |
| IL-2 | NIH AIDS Reagent Program, NIH from Dr. Maurice Gately, Hoffmann - La Roche Inc |  |
| Lipopolysaccharide (LPS) | Sigma-Aldrich | Cat#L2137 |
| Brefeldin A | Life Technologies | Cat#B7450 |
| CLI-095 | InvivoGen | Cat#243984-11-4 |
| Dimethyl Sulfoxide | Sigma-Aldrich | Cat#D8418 |
| Poly-L-Lysine | Fisher Scientific | Cat#11440812 |
| Bovine Serum Albumin (BSA) | GE Healthcare | Cat#SH30574 |
| DAPI Fluoromount-G® | Southern Biotech | Cat#0100-20 |
| Human Tenascin-C purified protein (TNC) | Merck Millipore | Cat#CC065 |
|  |  |  |

Critical commercial assays

| Anti-CCP ELISA (IgG) | EUROIMMUN | Cat#EA 1505-9601 G |
| --- | --- | --- |
| Human Tenascin-C Large (FNⅢ-C) Assay Kit - IBL | Immuno-Biological Laboratories Co., Ltd. | Cat#27751 |

Software

| BD FACSDiva™ | www.bdbiosciences.com | Version 8.0.1 |
| --- | --- | --- |
| FlowJo | www.flowjo.com | Version 10.7.1 |
| Pluggin: FlowAI | https://www.flowjo.com/exchange/#/ | Version 2.1 |
| Pluggin: DownSample | https://www.flowjo.com/exchange/#/ | Version 3.3 |
| GraphPad Prism | www.graphpad.com | Version 9.0.0 |
| IBM SPSS Statistic | www.ibm.com | Version 26 |
|  |  |  |
| Imaris | www.imaris.oxinst.com | Version 9.5.0 |
| Huygens Essential | www.svi.nl/Huygens-Software | Version 19.10 |
| Microsoft Excel | www.microsoft.com | Version 16.0 |
| Adobe Illustrator | www.adobe.com | Version 25.2 |
