# Supplementary material for: Direct tissue-sensing reprograms TLR4+ Tfh-like cells inflammatory profile in the joints of rheumatoid arthritis patients
Source: Commun Biol. 2021 Sep 27;4:1135. doi: 10.1038/s42003-021-02659-0 (PMC8476501; doi:10.1038/s42003-021-02659-0)
Supplement: Supplementary file 6 — Supplementary Data 3 [file 42003_2021_2659_MOESM6_ESM.docx]

Supplementary Data 3. Statistical summary per figure

|  | Statistical report | Sample size |
| --- | --- | --- |
| Figure 1 |  |  |
| a) | *M* = 25.3, *SD* = 19.1 | *n* = 12 RA patients |
| b) | *t* (11) = 7.05, *****p* < 0.0001, ^+++^*d* = 2.04 | *n* = 12 RA patients |
| c) | *t* (11) = 5.39, ****p* = 0.0002, ^+++^*d* = 1.56 | *n* = 12 RA patients |
| d) | *Mdn* = 3.16, *IQR* = 1.12-8.95 | *n* = 100 RA patients |
| e) | *Z* = 8.68, *****p* < 0.0001, ^+++^*r* = 0.61, *n* = 100 | *n* = 100 RA patients |
| f) | *Z* = 8.68, *****p* < 0.0001, ^+++^*r* = 0.61, *n* = 100 | *n* = 100 RA patients |
| g) | *t* (11) = 4.38, ***p* = 0.001, ^+++^*d* = 1.27 | *n* = 12 RA patients |
| h) | *Z* = 2.93, ****p* = 0.001, ^+++^*r* = 0.63 | *n* = 11 RA patients |
| i) | ^+++^*r_p_*(10) = 0.74, ***p* = 0.006 | *n* = 12 RA patients |
| l) | *U* = 665, *****p* < 0.0001, ^+++^*r* = 0.53 | *n* = 76 HLA-DR^-^ vs 47 HLA-DR^+^ CD4^+^ T cells |
| m) | *U* = 610, *****p* < 0.0001, ^+++^*r* = 0.55 | *n* = 76 HLA-DR^-^ vs 47 HLA-DR^+^ CD4^+^ T cells |
| n) | *U* = 380, *****p* < 0.0001, ^+++^*r* = 0.64 | *n* = 75 HLA-DR^-^ vs 44 HLA-DR^+^ CD4^+^ T cells |
| Figure 2 |  |  |
| a) | *t* (12) = 11.7, *****p* < 0.0001, ^+++^*d* = 3.25 | *n* = 13 RA patients |
| b) | *Z* = 3.18, ****p* = 0.0002, ^+++^*r* = 0.62 | *n* = 13 RA patients |
| c) | *Z* = 3.18, ****p* = 0.0002, ^+++^*r* = 0.62 | *n* = 13 RA patients |
| d) | *Z* = 3.18, ****p* = 0.0002, ^+++^*r* = 0.62 | *n* = 13 RA patients |
| Figure 3 |  |  |
| a) | *Mdn* = 8.48, *IQR* = 3.48-14.1 | *n* = 6 RA patients |
| b) | *t* (3) = 3.24, **p* = 0.048, ^+++^*d* = 1.12 | *n* = 4 RA patients |
| d) | χ*^2^*(2) = 70.0, *****p* < 0.0001, ^+++^η^2^ = 0.52  Dunn’s multiple comparisons posttest:  Unstimulated vs TCR CD28 - *****p* < 0.0001  Unstimulated vs TCR CD28 LPS - *****p* < 0.0001  TCR CD28 vs TCR CD28 LPS - ^ns^*p* = 0.67 | *n* = 43 unstimulated vs 40 TCR CD28 vs 50 TCR CD28 LPS cells from 4 different HD |
| e) | *Z* = 5.84, *****p* < 0.0001, ^+++^*r* = 0.57 | *n* = 53 independent experiments (HD cells) |
| f) | *t* (38) = 8.53, *****p* < 0.0001, ^+++^*d* = 1.37 | *n* = 39 independent experiments (HD cells) |
| g) | *Z* = 2.02, ^ns^*p* = 0.063, ^+++^*r* = 0.64 | *n* = 5 HD |
| h) | *Z* = 2.02, ^ns^*p* = 0.063, ^+++^*r* = 0.64 | *n* = 5 HD |
| Figure 4 |  |  |
| a) | *U* = 957, ^ns^*p* = 0.11, ^+^*r* = 0.16 | *n* = 64 ≤65 years vs 37 >65 years RA patients |
| b) | *U* = 457, ^ns^*p* = 0.073, ^+^*r* = 0.18 | *n* = 86 female vs 15 male RA patients |
| c) | *U* = 587, ^ns^*p* = 0.75, ^+^*r* = 0.036 | *n* = 65 RF^+^ vs 19 RF^-^ RA patients |
| d) | ^+^*r_s_*(63) = 0.069, ^ns^*p* = 0.59 | *n* = 65 RF^+^ RA patients |
| e) | *U* = 734, ^ns^*p* = 0.20, ^+^*r* = 0.13 | *n* = 71 CCP^+^ vs 25 CCP^-^ RA patients |
| f) | ^++^*r_s_*(69) = 0.36, ***p* = 0.002 | *n* = 71 CCP^+^ RA patients |
| g) | ^+^*r_s_*(79) = -0.088, ^ns^*p* = 0.43 | *n* = 81 RA patients |
| h) | ^+^*r_s_*(79) = -0.074, ^ns^*p* = 0.51 | *n* = 81 RA patients |
| i) | χ*^2^*(2) = 1.64, ^ns^*p* = 0.44, ^+^η^2^ = 0.004 | *n* = 8 NSAIDs and/or corticoids vs 80 DMARDs vs 13 biological DMARDs RA patients |
| j) | *U* = 119, ^ns^*p* = 0.78, ^+^*r* = 0.028 | *n* = 101 RA patients |
| k) | *U* = 909, ^ns^*p* = 0.49, ^+^*r* = 0.069 | *n* = 101 RA patients |
| l) | *U* = 780, **p* = 0.047, ^+^*r* = 0.20 | *n* = 101 RA patients |
| m) | *U* = 474, **p* = 0.015, ^+^*r* = 0.24 | *n* = 101 RA patients |
| n) | *U* = 371, ^ns^*p* = 0.61, ^+++^*r* = 0.52 | *n* = 101 RA patients |
| o) | *U* = 215, ^ns^*p* = 0.70, ^+^*r* = 0.040 | *n* = 101 RA patients |
| p) | *U* = 451, ^ns^*p* = 0.22, ^+^*r* = 0.12 | *n* = 101 RA patients |
| q) | ^+^*r_s_*(87) = 0.033, ^ns^*p* = 0.76 | *n* = 89 RA patients |
| Figure 5 |  |  |
| a) | *Mdn* = 3.54, *IQR* = 0.95-9.11 | *n* = 99 RA patients |
| b) | *Mdn* = 87.3, *IQR* = 75.5-92.9 | *n* = 99 RA patients |
| c) | *Mdn* = 99.4, *IQR* = 97.9-99.8 | *n* = 99 RA patients |
| d) | ^+^*r_s_*(97) = 0.23, **p* = 0.025 | *n* = 99 RA patients |
| e) | *Z* = 3.62, *****p* < 0.0001, ^+++^*r* = 0.62 | *n* = 17 RA patients |

| Figure 6 |  |  |
| --- | --- | --- |
| b) | *Z* = 3.18, ****p* = 0.0002, ^+++^*r* = 0.62 | *n* = 13 RA patients |
| c) | *Z* = 3.18, ****p* = 0.0002, ^+++^*r* = 0.62 | *n* = 13 RA patients |
| d) | *Z* = 3.18, ****p* = 0.0002, ^+++^*r* = 0.62 | *n* = 13 RA patients |
| e) | *Z* = 3.18, ****p* = 0.0002, ^+++^*r* = 0.62 | *n* = 13 RA patients |
| f) | *Z* = 3.18, ****p* = 0.0002, ^+++^*r* = 0.62 | *n* = 13 RA patients |
| g) | *t* (12) = 2.93, **p* = 0.013, ^+++^*d* = 0.81 | *n* = 13 RA patients |
| h) | *Z* = 2.27, **p* = 0.021, ^++^*r* = 0.45 | *n* = 13 RA patients |
| i) | *Z* = 3.18, ****p* = 0.0002, ^+++^*r* = 0.62 | *n* = 13 RA patients |
| j) | *Z* = 3.18, ****p* = 0.0002, ^+++^*r* = 0.62 | *n* = 13 RA patients |
| k) | ^+++^*r_s_*(11) = 0.69, ***p* = 0.009 | *n* = 13 RA patients |
| l) | ^+++^*r_s_*(11) = 0.70, ***p* = 0.007 | *n* = 13 RA patients |
| Figure 7 |  |  |
| b) | *Z* = 3.06, ****p* = 0.0005, ^+++^*r* = 0.62 | *n* = 12 RA patients |
| c) | *t* (11) = 9.42, *****p* < 0.0001, ^+++^*d* = 2.72 | *n* = 12 RA patients |
| d) | *t* (11) = 3.28, ***p* = 0.007, ^+++^*d* = 0.95 | *n* = 12 RA patients |
| e) | *Z* = 3.06, ****p* = 0.0005, ^+++^*r* = 0.62 | *n* = 12 RA patients |
| Figure 8 |  |  |
| c) | *Z* = 2.98, ****p* = 0.001, ^+++^*r* = 0.61 | *n* = 12 RA patients |
| d) | *Z* = 2.51, ***p* = 0.009, ^+++^*r* = 0.51 | *n* = 12 RA patients |
| e) | *Z* = 1.92, ^ns^*p* = 0.055, ^++^*r* = 0.38 | *n* = 13 RA patients |
| f) | *t* (12) = 0.61, ^ns^*p* = 0.55, ^+^*d* = 0.17 | *n* = 13 RA patients |
| g) | *Z* = 3.18, ****p* = 0.0002, ^+++^*r* = 0.62 | *n* = 13 RA patients |
| h) | *t* (12) = 8.95*, ******p* < 0.0001, ^+++^*d* = 2.48 | *n* = 13 RA patients |
| i) | *Z* = 2.34, **p* = 0.017, ^+++^*r* = 0.50 | *n* = 13 RA patients |
| j) | *Z* = 3.18, ****p* = 0.0002, ^+++^*r* = 0.62 | *n* = 13 RA patients |
| Figure 9 |  |  |
| a) | χ*^2^*(3) = 17.6, *****p* < 0.0001, ^+++^*W* = 0.84  Dunn’s multiple comparisons posttest:  Unstimulated vs TCR ICOS - **p* = 0.023  Unstimulated vs TCR ICOS LPS - ***p* = 0.003  Unstimulated vs LPS - ^ns^*p* > 0.99  TCR ICOS vs TCR ICOS LPS - ^ns^*p* > 0.99  TCR ICOS vs LPS - ^ns^*p* = 0.14  TCR ICOS LPS vs LPS - **p* = 0.023 | *n* = 7 RA patients |
| b) | χ*^2^*(3) = 12.6, ***p* = 0.002, ^+++^*W* = 0.60  Dunn’s multiple comparisons posttest:  Unstimulated vs TCR ICOS - ^ns^*p* = 0.078  Unstimulated vs TCR ICOS LPS - ***p* = 0.006  Unstimulated vs LPS - ^ns^*p* > 0.99  TCR ICOS vs TCR ICOS LPS - ^ns^*p* > 0.99  TCR ICOS vs LPS - ^ns^*p* > 0.99  TCR ICOS LPS vs LPS - ^ns^*p* = 0.23 | *n* = 7 RA patients |
| c) | *F*(3, 18) = 9.20, ****p* = 0.0007, ^+++^η_p_^2^ = 0.61; sphericity assumed  Tukey’s multiple comparisons posttest:  Unstimulated vs TCR ICOS - **p* = 0.042  Unstimulated vs TCR ICOS LPS - ***p* = 0.002  Unstimulated vs LPS - ****p* = 0.0009  TCR ICOS vs TCR ICOS LPS - ^ns^*p* = 0.47  TCR ICOS vs LPS - ^ns^*p* = 0.31  TCR ICOS LPS vs LPS - ^ns^*p* = 0.99 | *n* = 7 RA patients |
| d) | χ*^2^*(3) = 5.74, ^ns^*p* = 0.12, ^+^*W* = 0.27 | *n* = 7 RA patients |
| e) | χ*^2^*(3) = 13.1, ****p* = 0.0002, ^+++^*W* = 0.87  Dunn’s multiple comparisons posttest:  Unstimulated vs TCR ICOS - ^ns^*p* > 0.99  Unstimulated vs TCR ICOS LPS - ***p* = 0.009  Unstimulated vs LPS - ^ns^*p* = 0.086  TCR ICOS vs TCR ICOS LPS - ^ns^*p* = 0.086  TCR ICOS vs LPS - ^ns^*p* = 0.52  TCR ICOS LPS vs LPS - ^ns^*p* > 0.99 | *n* = 5 RA patients |
| f) | χ*^2^*(3) = 9.24, **p* = 0.017, ^+++^*W* = 0.62  Dunn’s multiple comparisons posttest:  Unstimulated vs TCR ICOS - ^ns^*p* > 0.99  Unstimulated vs TCR ICOS LPS - **p* = 0.042  Unstimulated vs LPS - ^ns^*p* = 0.85  TCR ICOS vs TCR ICOS LPS - ^ns^*p* = 0.086  TCR ICOS vs LPS - ^ns^*p* > 0.99  TCR ICOS LPS vs LPS - ^ns^*p* > 0.99 | *n* = 5 RA patients |
| g) | χ*^2^*(3) = 21.3, *****p* < 0.0001, ^+++^*W* = 0.59  Dunn’s multiple comparisons posttest:  Unstimulated vs TCR ICOS - ^ns^*p* = 0.055  Unstimulated vs TCR ICOS LPS - *****p* < 0.0001  Unstimulated vs LPS - ^ns^*p* = 0.49  TCR ICOS vs TCR ICOS LPS - ^ns^*p* = 0.35  TCR ICOS vs LPS - ^ns^*p* > 0.99  TCR ICOS LPS vs LPS - **p* = 0.033 | *n* = 12 RA patients |
| h) | χ*^2^*(3) = 7.90, **p* = 0.045, ^+^*W* = 0.22  Dunn’s multiple comparisons posttest:  Unstimulated vs TCR ICOS - ^ns^*p* = 0.68  Unstimulated vs TCR ICOS LPS - **p* = 0.043  Unstimulated vs LPS - ^ns^*p* > 0.99  TCR ICOS vs TCR ICOS LPS - ^ns^*p* > 0.99  TCR ICOS vs LPS - ^ns^*p* > 0.99  TCR ICOS LPS vs LPS - ^ns^*p* = 0.35 | *n* = 12 RA patients |
| Figure 10 |  |  |
| a) | ^+^*r_p_*(4) = 0.34, ^ns^*p* = 0.51 | *n* = 6 RA patients |
| b) | ^+^*r_p_*(5) = 0.12, ^ns^*p* = 0.80 | *n* = 7 RA patients |
| c) | ^+^*r_p_*(5) = 0.33, ^ns^*p* = 0.46 | *n* = 7 RA patients |
| d) | χ*^2^*(2) = 8.40, ***p* = 0.008, ^+++^*W* = 0.84  Dunn’s multiple comparisons posttest:  Unstimulated vs SF - **p* = 0.013  Unstimulated vs SF+CLI-095 - ^ns^*p* > 0.99  SF vs SF+CLI-095 - ^ns^*p* = 0.17 | *n* = 5 RA patients |
| e) | *F*(2, 8) = 4.86, **p* = 0.042, ^+++^η_p_^2^ = 0.55; sphericity assumed  Tukey’s multiple comparisons posttest:  Unstimulated vs SF - ^ns^*p* = 0.72  Unstimulated vs SF+CLI-095 - ^ns^*p* = 0.13  SF vs SF+CLI-095 - **p* = 0.040 | *n* = 5 RA patients |
| f) | χ*^2^*(2) = 2.80, ^ns^*p* = 0.37, ^+^*W* = 0.28 | *n* = 5 RA patients |
| g) | χ*^2^*(2) = 0.40, ^ns^*p* = 0.95, ^+^*W* = 0.040 | *n* = 5 RA patients |
| h) | *t* (5) = 1.88*,* ^ns^*p* = 0.12, ^++^*d* = 0.77 | *n* = 6 RA patients |
| i) | *t* (4) = 0.82*,* ^ns^*p* = 0.46, ^++^*d* = 0.37 | *n* = 5 RA patients |
| j) | *t* (4) = 1.81*,* ^ns^*p* = 0.15, ^+++^*d* = 0.81 | *n* = 5 RA patients |
| k) | *Z* = 1.21, ^ns^*p* = 0.31, ^++^*r* = 0.38 | *n* = 5 RA patients |
| Supp. Fig. 5 |  |  |
| a) | *t* (9) = 3.11*,* **p* = 0.013, ^+++^*d* = 0.98 | *n* = 10 RA patients |
| b) | *t* (9) = 4.57*, ****p* = 0.001, ^+++^*d* = 1.50 | *n* = 10 RA patients |
| c) | *t* (9) = 3.50*, ****p* = 0.007, ^+++^*d* = 1.11 | *n* = 10 RA patients |
| d) | *t* (9) = 3.55*, ****p* = 0.006, ^+++^*d* = 1.12 | *n* = 10 RA patients |
| e) | *Z* = 1.99, ^ns^*p* = 0.063, ^+++^*r* = 0.58 | *n* = 6 RA patients |
| f) | *t* (5) = 4.68*, ****p* = 0.005, ^+++^*d* = 1.91 | *n* = 6 RA patients |
| g) | *Z* = 2.20, **p* = 0.031, ^+++^*r* = 0.64 | *n* = 6 RA patients |
| h) | *t* (5) = 0.35*,* ^ns^*p* = 0.74, ^+^*d* = 0.14 | *n* = 6 RA patients |
| i) | *Z* = 1.36, ^ns^*p* = 0.22, ^++^*r* = 0.39 | *n* = 6 RA patients |
|  |  |  |
