# Supplementary material for: Direct tissue-sensing reprograms TLR4+ Tfh-like cells inflammatory profile in the joints of rheumatoid arthritis patients
Source: Commun Biol. 2021 Sep 27;4:1135. doi: 10.1038/s42003-021-02659-0 (PMC8476501; doi:10.1038/s42003-021-02659-0)
Supplement: Supplementary file 18 — Reporting Summary [file 42003_2021_2659_MOESM18_ESM.pdf]

nature portfolio

Corresponding author(s): DBPR COMMSBIO-21-0335A

Last updated by author(s): Aug 5, 2021

Reporting Summary

Nature Portfolio wishes to improve the reproducibility of the work that we publish. This form provides structure for consistency and transparency in reporting. For further information on Nature Portfolio policies, see our Editorial Policies and the Editorial Policy Checklist.

Statistics

For all statistical analyses, confirm that the following items are present in the figure legend, table legend, main text, or Methods section.

n/a	Confirmed	
☐	☒	The exact sample size (*n*) for each experimental group/condition, given as a discrete number and unit of measurement
☐	☒	A statement on whether measurements were taken from distinct samples or whether the same sample was measured repeatedly
☐	☒	The statistical test(s) used AND whether they are one- or two-sided<br>*Only common tests should be described solely by name; describe more complex techniques in the Methods section.*
☐	☒	A description of all covariates tested
☐	☒	A description of any assumptions or corrections, such as tests of normality and adjustment for multiple comparisons
☐	☒	A full description of the statistical parameters including central tendency (e.g. means) or other basic estimates (e.g. regression coefficient) AND variation (e.g. standard deviation) or associated estimates of uncertainty (e.g. confidence intervals)
☐	☒	For null hypothesis testing, the test statistic (e.g. F, t, r) with confidence intervals, effect sizes, degrees of freedom and P value noted<br>*Give P values as exact values whenever suitable.*
☒	☐	For Bayesian analysis, information on the choice of priors and Markov chain Monte Carlo settings
☒	☐	For hierarchical and complex designs, identification of the appropriate level for tests and full reporting of outcomes
☐	☒	Estimates of effect sizes (e.g. Cohen's d, Pearson's r), indicating how they were calculated

Our web collection on statistics for biologists contains articles on many of the points above.

Software and code

Policy information about availability of computer code

Data collection	Flow Cytometry data was collected using BD FACSDiva™ www.bdbiosciences.com Version 8.0.1 and microscopy data was collected using Huygens Essential www.svi.nl/Huygens-Software Version 19.10.
Data analysis	For Flow Cytometry analysis it was used BD FACSDiva™ www.bdbiosciences.com Version 8.0.1, FlowJo www.flowjo.com Version 10.7.1 with Pluggin: FlowAI https://www.flowjo.com/exchange/#/ Version 2.1 and Pluggin: DownSample https://www.flowjo.com/exchange/#/ Version 3.3. For microscopy data it was used Imaris www.imaris.oxinst.com Version 9.5.0. For cumulative analysis it was used GraphPad Prism www.graphpad.com Version 9.0.0, IBM SPSS Statistic www.ibm.com Version 26 and Microsoft Excel www.microsoft.com Version 16.0. Then panels were composed by using Adobe Illustrator www.adobe.com Version 25.2.

For manuscripts utilizing custom algorithms or software that are central to the research but not yet described in published literature, software must be made available to editors and reviewers. We strongly encourage code deposition in a community repository (e.g. GitHub). See the Nature Portfolio guidelines for submitting code & software for further information.

Data

Policy information about availability of data

All manuscripts must include a data availability statement. This statement should provide the following information, where applicable:

- Accession codes, unique identifiers, or web links for publicly available datasets
- A description of any restrictions on data availability
- For clinical datasets or third party data, please ensure that the statement adheres to our policy

The datasets generated during and/or analyzed during the current study are available from the corresponding author on reasonable request.

Field-specific reporting

Please select the one below that is the best fit for your research. If you are not sure, read the appropriate sections before making your selection.

☒ Life sciences ☐ Behavioural & social sciences ☐ Ecological, evolutionary & environmental sciences

For a reference copy of the document with all sections, see nature.com/documents/nr-reporting-summary-flat.pdf

Life sciences study design

All studies must disclose on these points even when the disclosure is negative.

Sample size	No statistical methods were used to calculate the sample size. We used a convenience sample, whose size was determined based on the number of patients consulted by the rheumatology service of Hospital Egas Moniz, Lisbon from 2017 to July 2021 that were enrolled and provided consent to the study under NMS and HEM approved ethics protocols 84/2019/CEFCM and 20170700050, respectively. In total, 120 RA patients and 29 HD controls were included in this study.
Data exclusions	One RA donor was excluded for intracellular cytokine production, as this individual had a population of interest inferior to 0.3%. The low event number precluded a robust analysis of cytokine production. Data from this donor was excluded from intracellular cytokine production analyses.
Replication	The experiments were not replicated- cross-sectional analyses of samples from humans.
Randomization	Randomization was not relevant to this study, as this is an observational study. Participants were chosen based on their presence to consultations and consent to participate in the study.
Blinding	As the samples were obtained from hospital with a code number and without any identifying information, at the time of sample acquisition and processing the investigator was blind to patients' group allocations. Patients' clinical information and scores were only revealed after data collection.

Reporting for specific materials, systems and methods

We require information from authors about some types of materials, experimental systems and methods used in many studies. Here, indicate whether each material, system or method listed is relevant to your study. If you are not sure if a list item applies to your research, read the appropriate section before selecting a response.

Materials & experimental systems

n/a	Involved in the study	
☐	☒	Antibodies
☒	☐	Eukaryotic cell lines
☒	☐	Palaeontology and archaeology
☒	☐	Animals and other organisms
☐	☒	Human research participants
☒	☐	Clinical data
☒	☐	Dual use research of concern

Methods

n/a	Involved in the study	
☒	☐	ChIP-seq
☐	☒	Flow cytometry
☒	☐	MRI-based neuroimaging

Antibodies

Antibodies used	All primary antibodies used are against human proteins<br>Anti-hCD3 (UCHT1) BioLegend Cat#300402<br>Anti-hHLA-DR (L243) BioLegend Cat#307602<br>Anti-hICOS (C398-4A) BioLegend Cat#313512<br>Anti-hTLR4 (HTA125) BioLegend Cat#312804<br>Anti-hTLR4 (76B357.1) Abcam Cat#ab22048<br>Anti-hIL1R (C-20) Santa Cruz Cat#sc-687<br>Anti-hCD28 (CD28.2) BioLegend Cat#302914<br>Anti-hCD4 (RPA-T4) BioLegend Cat#300506 (FITC)<br>Anti-hHLA-DR (L243) BioLegend Cat#307606 (PE)<br>Anti-hTLR4 (HTA125) BioLegend Cat#312805 (PE)<br>Anti-hTNF-α (MAb11) Biolegend Cat#502926 (PerCP/Cy5.5)<br>Anti-hPD1 (EH12.2H7) BioLegend Cat#329917 (PeCy7)<br>Anti-hCCR2 (K036C2) BioLegend Cat#357211 (PeCy7)

	Anti-hCD25 (M-A251) BioLegend Cat#356107 (PeCy7)<br>Anti-hKi67 (B56) BD Pharmigen Cat#561283 (PeCy7)<br>Anti-hTNFa (MAb11) BioLegend Cat#502929 (PeCy7)<br>Anti-hIL-10 (JES3-9D7) BioLegend Cat#501419 (PE-Cy7)<br>Anti-hCD4 (RPA-T4) BioLegend Cat#300514 (APC)<br>Anti-hIL6R (UV4) BioLegend Cat#352805 (APC)<br>Anti-hICOS (C398.4A) BioLegend Cat#313510 (APC)<br>Anti-hIL17R (BG/hIL17AR) BioLegend Cat#340903 (A647)<br>Anti-hIL-21 (3A3-N2) BioLegend Cat#513006 (A647)<br>Anti-hCD3 (HIT3a) BioLegend Cat#300318 (APC-Cy7)<br>Anti-hCCR6 (G034E3) BioLegend Cat#353432 (APC-Cy7)<br>Anti-hCD38 (HIT2) BioLegend Cat#303533 (APC-Cy7)<br>Anti-hCXCR5 (J252D4) BioLegend Cat#356925 (APC-Cy7)<br>Anti-hIL17 (BL168) Biolegend Cat#512320 (APC-Cy7)<br>Anti-hCD14 (63D3) BioLegend Cat#367107 (APC-Cy7)<br>Anti-hCD3 (SK7) BioLegend Cat#344828 (Bv510)<br>Anti-hCD3 (SK7) BioLegend Cat#3448284 (PB)<br>Anti-hIFN-γ (4S.B3) BioLegend Cat#502522 (PB)<br>Anti-hCD4 (SK3) BioLegend Cat#344666 (PE-Fire 700)<br>Anti-hCD3 (UCHT1) BioLegend Cat#300424 (A700)<br>Anti-hCD14 (63D3) BioLegend Cat#367125 (Bv605)<br>Anti-hIL-10 (JES3-19F1) BioLegend Cat#506811 (PE-Dazzle 594)<br><br>Secondary antibodies used<br>Anti-mouse IgG2b Thermo Fisher Cat#A21141 (A488)<br>Anti-rabbit Invitrogen Cat#A-21244 (A647)<br>Anti-mouse IgG1 Thermo Fisher Cat#A21240 (A647)<br>Anti-mouse IgG1 BioLegend Cat#406602<br>Anti-hamster IgG Thermo Fisher Scientific Cat#31115
Validation	All antibodies used have been validated by the manufacturer and used according to the manufacturer's instruction. By searching the catalog number in the manufacturer's websites, the validation statement and all relevant information's including citations of each antibody listed above can be found. We have titrated these antibodies have according to our staining conditions. FMO were included in the experiments whenever appropriate.

Human research participants

Policy information about studies involving human research participants

Population characteristics	One-hundred and twenty one RA patients fulfilling the ACR 2010 classification criteria and thirty healthy controls were included. The details of the RA cohort and healthy volunteers involved in the study are summarised in Supplementary Table 1 and Supplementary Table 2, respectively.
Recruitment	Participants were recruited based on their attendance at routine consultations in the rheumatology service of Hospital Egas Moniz, between 2017 and July 2021 and willingness to participate in the study. Informed consent was obtained by trained staff and sample collection commenced immediatelt upon study enrollment.
Ethics oversight	The Ethics Committee of NOVA Medical School (84/2019/CEFCM) and of Hospital Egas Moniz (20170700050) approved this study.

Note that full information on the approval of the study protocol must also be provided in the manuscript.

Flow Cytometry

Plots

Confirm that:

- [x] The axis labels state the marker and fluorochrome used (e.g. CD4-FITC).
- [x] The axis scales are clearly visible. Include numbers along axes only for bottom left plot of group (a 'group' is an analysis of identical markers).
- [x] All plots are contour plots with outliers or pseudocolor plots.
- [x] A numerical value for number of cells or percentage (with statistics) is provided.

Methodology

Sample preparation	Blood samples and synovial fluid were processed within 4 hours of collection and freshly analyzed. Peripheral blood and synovial mononuclear cells were isolated by density gradient centrifugation (Biocoll, Merck Millipore) or following enzymatic digestion with hyaluronidase (10µL/mL; 30min at 37ºC), respectively. Plasma and cell-depleted synovial fluid were frozen until further use.

	For flow cytometry analysis peripheral blood cells were stained with antibodies listed in Supplementary Table 3. For cell viability, Fixable Viability Dye (eBioscience) or Calcein Violet-AM (Biolegend) were used. For intracellular staining cells were treated with Transcriptional Factor Fixation/Permeabilization kit (ebioscience). For cytokine experiments cells were fixed in paraformaldehyde 1% (Sigma-Aldrich) and permeabilized with saponin (Carl Roth).
Instrument	FACS acquisition was performed in a BD FACSCanto II instrument (BD Biosciences), BD FACSAria III (BD Biosciences).
Software	To collect Flow Cytometry data, BD FACSDiva™ www.bdbiosciences.com Version 8.0.1 was used. For Flow Cytometry Analysis it was used: BD FACSDiva™ www.bdbiosciences.com Version 8.0.1 and FlowJo www.flowjo.com Version 10.7.1 with Pluggin: FlowAI https://www.flowjo.com/exchange/#/ Version 2.1 and Pluggin: DownSample https://www.flowjo.com/exchange/#/ Version 3.3.
Cell population abundance	Cell population abundance: cell populations were reported as a proportion of live, or as a proportion of a parent gate (% of CD4 T cells, etc). The full gating strategy is included both in main and in supplementary figures
Gating strategy	After all abnormal events would be excluded by using FlowAI, SSC-A and FSC-A parameters were used to select leukocytes from isolated PBMCs. Live-dead markers were used to exclude dead cells. FSC-W/FSC-A parameters were used to exclude doublets. CD4+T cells were gated based on their markers (CD3, CD4), activated T cells were defined using HLA-DR, PD-1, CD38, Ki-67, Tfh cells were defined using CXCR5, PD1 and ICOS, migration pattens were defined using CCR2, CCR6 and CXCR5, inflammatory profile was determined using IL-1R, IL-2R, IL-6R and IL-17R, TLR4 T cells were defined using TLR4. Intracellular T cell gating to identify TLR4 positive and negative T cells producing IFN-g, IL-17, IL-21 and/or TNF-a were defined using the specific markers CD3, CD4, FN-g, IL-17, IL-21 and TNF-a.

☒ Tick this box to confirm that a figure exemplifying the gating strategy is provided in the Supplementary Information.
